# Multiple Simulated Annealing-Molecular Dynamics (MSA-MD) for Conformational Space Search of Peptide and Miniprotein

**DOI:** 10.1038/srep15568

**Published:** 2015-10-23

**Authors:** Ge-Fei Hao, Wei-Fang Xu, Sheng-Gang Yang, Guang-Fu Yang

**Affiliations:** 1 Key Laboratory of Pesticide & Chemical Biology, Ministry of Education, College of Chemistry, Central China Normal University, Wuhan 430079, P.R.China; 2 Collaborative Innovation Center of Chemical Science and Engineering, Tianjing 300072, P.R.China

## Abstract

Protein and peptide structure predictions are of paramount importance for understanding their functions, as well as the interactions with other molecules. However, the use of molecular simulation techniques to directly predict the peptide structure from the primary amino acid sequence is always hindered by the rough topology of the conformational space and the limited simulation time scale. We developed here a new strategy, named Multiple Simulated Annealing-Molecular Dynamics (MSA-MD) to identify the native states of a peptide and miniprotein. A cluster of near native structures could be obtained by using the MSA-MD method, which turned out to be significantly more efficient in reaching the native structure compared to continuous MD and conventional SA-MD simulation.

Protein and peptide tertiary structures are of paramount importance for understanding their function, as well as the interactions with other molecules. Peptide plays many biological functions such as hormones, neurotransmitters to antibiotics and so on. In addition, the folding mechanism also gives much more insight into the function of protein or peptide. However, considering the number of new sequences that are delivered by each genome project, present estimates of the number of hypothetical peptide code sequences in the complete prokaryotic genomes available today are on the order of 1.5 million, which is much higher in eukaryotes^1^,^2^. Hence, the majority of protein or peptide structures have not been resolved. Moreover it is hard to perform experimental study of the folding mechanism. To uncover the mystery of how proteins or peptides folding, molecular simulation techniques, as complementarities with experimental methodology, are frequently used for prediction and optimization of protein or peptide structures^3^.

The molecular simulation techniques for protein or peptide structure prediction can be divided into comparative modeling and *ab initio* prediction. The 3D structure of a protein can be predicted through comparative modeling based on the amino acid sequence and X-ray crystal structures of proteins with more than 30% sequence identity^4^. But without human intervention, comparative models result in low-accuracy due to errors as a result of inaccurate sequence alignment, and inability to identify and correctly model domains, such as loop and ligand-binding regions^5^. Even the proteins with high sequence identity may have different native structures^6^. In addition, compared with larger proteins, one major obstacle in predicting peptide structures is the limited number of solution structures are available^7^. *Ab initio* protein or peptide structure prediction refers to an algorithmic process by which protein tertiary structure is predicted from its amino acid primary sequence. The problem itself has occupied leading scientists for decades, which remains one of the top outstanding issues in modern science.

At present, some of the most successful *ab initio* methods have a reasonable probability of predicting the folds of small, single-domain proteins within 1.5 angstroms over the entire structure. For example, using MD simulation to perform protein or peptide structure prediction is one of the main types of *ab initio* methods, which include conventional molecular dynamics (CMD), simulated annealing molecular dynamics (SA-MD), replica exchange molecular dynamics (REMD) and some other methods through adding new algorithms in the above mentioned MD simulation^8^,^9^,^10^,^11^,^12^,^13^. Carlos Simmerling *et al.* has successfully predicted a “Trpcage” protein by using CMD method^14^, but the currently possible time scales still limit the sampled conformational space of biomolecules. Hence, Sugita *et al.* developed REMD method which can overcome the multiple-minima problem by simulating several replicas independently and simultaneously exchanging non-interacting replicas (neighboring pairs) of the system by performing CMD at several temperatures to obtain good prediction^15^. However, it need to parallel a lot of replicas simultaneously in order to get better overlap between the neighboring energy^16^, which needs relative high computational demands. To enhance conformational sampling, SA algorithm is to start the simulation at high temperature to overcome barriers followed by gradual cooling (annealing) to reach low energy regimes^17^. It is widely used for the optimization of structures from experimental methods^18^,^19^, comparative protein modeling^20^,^21^, or studying the conformational dynamics of protein or peptide folding and unfolding^22^.

In this work, we developed a strategy called Multiple Simulated Annealing-Molecular Dynamics (MSA-MD) which is a highly accurate prediction method combined Simulated Annealing-Molecular Dynamics (SA-MD) and empirical based screening for peptides. Based on MSA-MD, we can detect a wider conformational space of a peptide or miniprotein through large scale structure sampling. And the near native conformations of the pepides and proteins can be obtained. A conformational ensemble which is close to the protein native crystal structure can be obtained. This strategy is applied for the structure prediction of ALPHA1, Trp-cage protein, PolyAla, two pepides containing β sheet structure and two miniproteins containing more than 40 residues. Good ability in sampling lower energy conformations and wider conformation space were obtained. Figure 1 shows the prediction process of MSA-MD for small peptides in details. Two key issues were studied in this work: the conformation sampling (the capability of MSA-MD in searching of conformational space) and the conformation screening (how to screen the near native states).

## Results and Discussion

We evaluated the capability of MSA-MD in folding seven small peptides and proteins, ALPHA1 (PDB code:1AL1), Trp-cage miniprotein (PDB code:1L2Y), PolyAla, two pepides containing β sheet structures (PDB code:1UAO and 1E0Q) and two miniproteins containing more than 40 residues (PDB code:1ERD and 1GAB). The structural similarity was assessed by the root-mean-square deviation (RMSD) of the aligned Cα atom excluding the flexible termini, which is a similar treatment in other studies^23^. Starting from extended conformations, a total of 1000 simulated annealing MD simulations (each 10 ns) were performed for each protein (Table 1).

### The conformation sampling

#### ALPHA1

**Figure 2:**
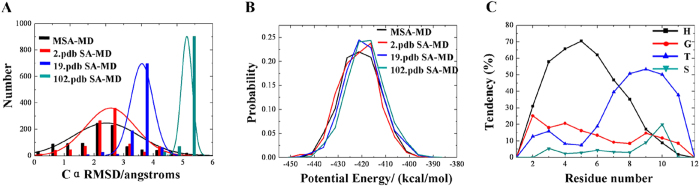
(**A**) The comparison of the Cα RMSD distribution of the structures generated by MSA-MD and SSA-MD. Black represents structures from 10 ns MSA-MD; red, blue and grey represent structures from the single trajectory of 2.pdb, 19.pdb, 102.pdb respectively. The Cα RMSD of 2.pdb, 19.pdb, 102.pdb relative to the 1AL1 crystal structure is 1.195 Å, 3.882 Å, 5.085 Å respectively. (**B**) The comparison of the potential energy distribution of the structures generated by MSA-MD and SSA-MD. (**C**) Secondary-structure-forming tendencies of the ALPHA1 as a function of residue number. The results are mean values over the final 1000 structures from 10 ns MSA-MD. Square with black solid line: alpha helix structure (H); circles with red solid line: 3-helix structure (G); up triangles with blue solid line: hydrogen bonded turn (T); down triangles with grey solid line: bend structure (S).

The sequence of ALPHA1 is ELLKKLLEELKG. In order to compare the performance of the MSA-MD and single simulated annealing-MD (SSA-MD), the performance of the conformational search were compared. Three SSA-MD trajectories were selected as representatives and compared with MSA-MD. The Cα RMSD of the 1000 structures from MSA-MD distributed in around 6 Å range with 2.5 Å as the maximum normal distribution. While in each SSA-MD, the Cα RMSD distributions were much narrower and the maximum normal distributions were much larger than MSA-MD (Fig. 2A). In addition, the maximum normal distributions of the potential energies were slightly larger in SSA-MD. But, no significant differences were found for the distribution range of the potential energy (Fig. 2B). Moreover, the MSA-MD was also compared with simulated annealing coupled replica exchange molecular dynamics (SA-REMD) developed by Kannan *et al.*^24^. The Cα RMSD distributed from 1 to 5 Å and the potential energies distributed from −420 to −340 kcal/mol in the SA-REMD. While, the range of Cα RMSD is from 0 to 6 Å and the range of potential energy is from −445 to −385 kcal/mol in MSA-MD. Hence, the MSA-MD can search a wider conformational space than SSA-MD and SA-REMD.

**Figure 3:**
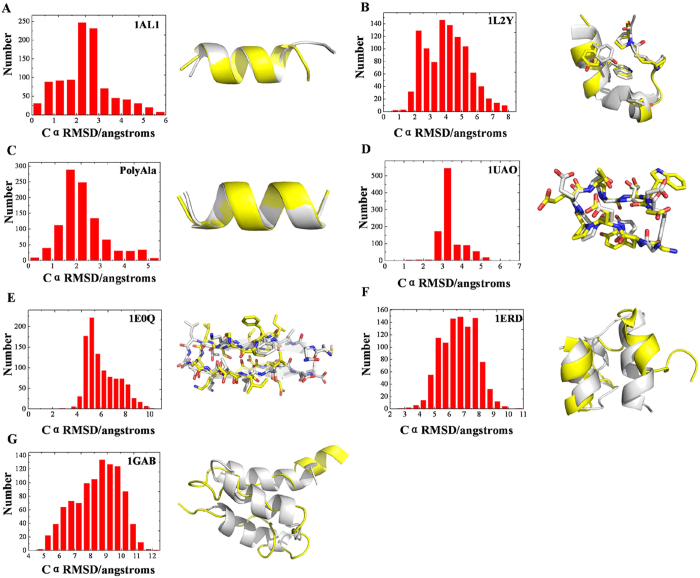
The Cα RMSD distributions of the structures generated by MSA-MD and the structural alignments with the native structure. The structures formed by the 10 ns MSA-MD compared with their own native structures of the seven peptides. White structure is the native conformation, and the yellow structure is the best structure formed by MSA-MD. (**A**) The Cα RMSD distribution of the structures formed by MSA-MD simulations relative to the ALPHA1 crystal structure and the overlay of the best predicted structure with the ALPHA1 native structure. (**B**) The Cα RMSD distribution of the structures formed by MSA-MD simulations relative to the 1L2Y crystal structure and the overlay of the best predicted structure with the 1L2Y native structure. (**C**) The Cα RMSD distribution of the structures formed by MSA-MD simulations relative to the PolyAla crystal structure and the overlay of the best predicted structure with the standard conformation. (**D**) The Cα RMSD distribution of the structures formed by MSA-MD simulations relative to the 1UAO crystal structure and the overlay of the best predicted structure with the 1UAO native structure. (**E**) The Cα RMSD distribution of the structures formed by MSA-MD simulations relative to the 1E0Q crystal structure and the overlay of the best predicted structure with the 1E0Q native structure. (**F**) The Cα RMSD distribution of the structures formed by MSA-MD simulations relative to the 1ERD crystal structure and the overlay of the best predicted structure with the 1ERD native structure. (**G**) The Cα RMSD distribution of the structures formed by MSA-MD simulations relative to the 1GAB crystal structure and the overlay of the best predicted structure with the 1GAB native structure.

To investigate the folding pattern of the simulated structures, the forming tendency of the secondary structure were analyzed for the 1000 structures by using DSSP program^25^ and compared with the ALPHA1 native secondary-structure. The forming tendency of alpha helix (H), 3-helix (G), hydrogen bonded turn (T), and bend (S) structure as a function of residue number were shown in Fig. 2C. The helix-forming tendency is dominant over other conformations and β-sheet forming tendency was not observed for ALPHA1. Hence, the secondary structure forming tendency is consistent with the native secondary structure of ALPHA1. In addition, there are 120 structures with Cα RMSD lower than 1.0 Å and 306 structures with Cα RMSD lower than 2.0 Å (Fig. 3A). There is one structure with Cα RMSD value = 0.198 Å compared with the native structure (Fig. 3A). Hence, a cluster of near native structures of ALPHA1 can be predicted by MSA-MD.

### Trp-cage protein

A more challenging protein with 20-residues was used to assess the performance of MSA-MD method^26^. The sequence of the Trp-cage protein is NLYIQWLKDGGPSSGRPPPS. This miniprotein can fold fast to a globular structure in solution (~4.1 μs)^27^, which consists of an α helix (residues 1–9), a short 3_10_ helix (residues 11–13), and coil. The terminal amino group and carboxylate group between the side chains of Asp9 and Arg16 formed a salt bridge and then stabilized the two hydrophobic cores that pack against each other, namely the residue 1–9 that form a helix and the residue 16–20 that form a loop. The small size and fast folding nature of Trp-cage miniprotein makes it an ideal test model to validate novel structural prediction method^28^,^29^,^30^,^31^,^32^.

To search a wider conformational space, Trp-cage miniprotein was simulated for 1000 trajectories (10 ns each) from extended initial structure by setting different random number. Figure 3B shows the Cα RMSD distribution of the 1000 structures from the trajectories, which ranges around 8 Å and covers a wide conformational space. There are 37 structures with Cα RMSD < 2.0 Å and 267 structures with Cα RMSD < 3.0 Å. The Cα RMSD value of the nearest native structure predicted by MSA-MD is 0.96 Å (As show in Fig. 3B).

### PolyAla

We also evaluate the ability of MSA-MD in folding PolyAlanine [Ac-(Ala)11-NH2] to the known α-helical structure^33^. Starting from extended conformation, 1000 folding simulations of PolyAla were performed respectively for 10 ns by setting different random number. Just like other studies to evaluate the simulation result of PolyAla by helicity^10^. We construct a fully helical PolyAla conformation as a standard conformation. Then we assessed the simulation result by Cα RMSD between the predicted structures and a constructed helical conformation. Figure 3C shows the Cα RMSD distribution of the 1000 structures formed by MSA-MD, which ranges around 5.5 Å. There are 49 structures with Cα RMSD < 1.0 Å and 450 structures with Cα RMSD < 2.0 Å. The lowest Cα RMSD value is 0.197 Å, which means MSA-MD can accurately predict the PolyAla structure (Fig. 3C).

### β sheet structure

To validate the prediction ability of MSA-MD for β sheet structure, such as β-hairpin or β-turn, two small proteins were tested. The first is a β-turn protein (PDB code:1UAO) which can form chignolin peptide^34^. The sequence is GYDPETGTWG. 1000 structures were obtained by performing MSA-MD simulations (each for 10 ns). The Cα RMSDs of the 1000 structures range around 6 Å (shown in Fig. 3D), which represent a wider conformational space. The lowest Cα RMSD of the structures predicted by MSA-MD relative to 1UAO is 1.200 Å. The overlay with 1UAO is shown in Fig. 3D. In addition, another β-hairpin protein (PDB code:1E0Q) contain 17 residues was also tested^35^. The sequence is MQIFVKTLDGKTITLEV and 1000 structures were obtained by performing MSA-MD. The range of Cα RMSD is around 9 Å (shown in Fig. 3E). The lowest Cα RMSD of the structures predicted by MSA-MD relative to 1E0Q is 2.955 Å with the overlaid structure in Fig. 3E. The peptides containing β sheet structure did not fold well to native conformation by performing MSA-MD simulation. It is because the secondary structure propensities observed in protein simulations depend heavily on the force field parameters used^36^. Many previous studies revealed the helix-favoring bias in the AMBER ff94 and ff99 force fields using an explicit solvent model or the generalized Born implicit solvent model^37^. Our simulation with simulated annealing algorithm at high temperatures cannot solve the force-field bias in folding study. The results imply that the intrinsic secondary structure bias in a force field cannot easily be solved by modifying parameters of simulation. Hence, one should consider the integrative effects of all the force field parameters to improve the secondary structure balance of a force field^38^. If the force-field bias can be resolved, MSA-MD will still be accurate for structure prediction of β sheet structure.

### Miniprotein with more residues

We also access the prediction ability of MSA-MD for two miniproteins with more residues. The first one (PDB code:1ERD) is a α-helix protein with 40 residues^24^. The sequence of 1ERD is: XDPMTCEQAMASCEHTMCGYCQGPLY MTCIGITTDPECGLP. And the second (PDB code: 1GAB) is a 53-residues protein containing α-helix structure^24^. The sequence is: TIDQWLLKNAKEDAIAELKKA GITSDFYFNAINKAKTVEEVNALKNEILKAHA. 1000 structures were obtained for both 1ERD and 1GAB by performing MSA-MD simulation. We calculated the Cα RMSD against the 1ERD structure with residues 1 to 3 and 35 to 40 excluded as flexible termini. The range of Cα RMSD is around 8 Å (Fig. 3F). The structure with the lowest Cα RMSD relative to 1ERD is 2.908 Å (Fig. 3F). Similar with 1ERD, we calculated the Cα RMSD against the 1GAB structure with residues 1 to 8 and 51 to 53 excluded as flexible termini. The range of Cα RMSD is around 8 Å (Fig. 3G). The Cα RMSD of the best predicted structure relative to 1GAB is 4.715 Å (Fig. 3G). MSA-MD can have a better effect for small peptides than miniproteins containing more than 40 residues, which is because the secondary structure propensities observed in protein simulations depend heavily on the force field parameters and sufficient sampling time. In short, to the tested seven peptides and miniproteins, MSA-MD can search lower energy conformations and wider conformation space.

### The conformation screening

The MSA-MD can search a large number of near-native conformations for the peptides containing α-helix structure. Hence, a screening strategy should be developed to choose the right conformation which is similar to the native structure. In this paper, we tested an empirical-based screening strategy to discover a conformational cluster near to the native structure.

#### ALPHA1

**Figure 4:**
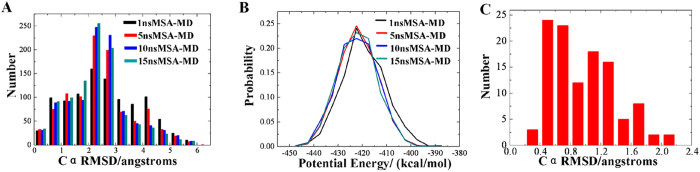
The Cα RMSD and the potential energy distribution of the 1000 structures extracted from 1, 5, 10, and 15 ns MSA-MD trajectory. (**A**) The Cα RMSD distribution of 1, 5, 10, and 15 ns MSA-MD simulations compared to 1AL1 crystal structure. (**B**) The potential energy distribution of 1, 5, 10, and 15 ns MSA-MD simulations. (**C**) The distribution of Cα RMSD value of the final 113 structures after three steps screen. All of the 113 structures have a Cα RMSD <2.2 Å, and 62 structures have a Cα RMSD <1.0 Å.

In order to examine the convergence of the simulated annealing, the potential energy distribution of the final structures from 1, 5, 10, and 15 ns were compared. In some other studies of structure prediction, Cα RMSD value lower than 3 Å is an acceptable minimum similarity for the small peptide^39^,^40^. As shown in Fig. 4A, the number of the structures with Cα RMSD smaller than 3 Å tends to be increasing when the simulation time is less than 10 ns. In addition, the potential energy distribution of the final structures from 1, 5, 10, and 15 ns were also compared. As shown in Fig. 4B, potential energy distribution move to the direction of lower potential energy from 1 to 5 ns. Hence, extension of the simulation time from 1 to 5 ns could improve the convergence of simulation. However, there is no significant difference of the potential energy distribution of 5, 10, and 15 ns simulations. Taking both the distribution of Cα RMSD and potential energy into account, choosing the time scale of 10 ns can get a good performance for the structure prediction of ALPHA1.

How to obtain the conformational ensemble similar with the native state of ALPHA1? To solve the problem, an empirical-based screening strategy to discover structures which are more similar with the native structure was proposed. First, the stereochemical qualities of 1000 predicted protein structures were evaluated by using PROCHECK program^41^,^42^ and the first nearly 20% structures (221 structures of the total 1000 structures with residues in most favored regions more than 90%) were screened out. Then, 221 structures were further clustered according to a threshold RMSD of 1.5 Å using MaxCluster program. The largest cluster of 132 structures is reserved, which occupy 59.73% of the total. Due to the flexibility, the difference of the RMSD value of the terminal residue may disturb the cluster. In order to strike off this influence, a new cluster analysis to the above 132 structures was performed. In this step, the RMSD values of the terminal residues were not taken into account and a threshold RMSD of 1.0 Å was used. All the 132 structures were further classified into 3 clusters with 113 structures in the largest cluster, which is 85.61% of the total.

In order to know that if the most structures obtained by three steps screening were similar with the native structure, the Cα RMSD of the final 113 structures compared to the native structure were statistically analyzed. The Cα RMSD < 1.0 Å and < 2.0 Å is 54.87% and 98.23% respectively, while the ratio is 49.24% and 97.73% before the third step, which demonstrate that most structures are similar with the native structure and the three-steps screening is reasonable. Figure 4C shows the distribution of Cα RMSD value of the final 113 structures. Based on the overlay between the best structure (No.964) and the crystal structure of ALPHA1, the Cα RMSD value of No.964 is 0.294 Å while its heavy atoms RMSD is 0.901 Å, which is lower than 1.3 Å (heavy atoms RMSD), the best structure predicted by SA-REMD previously^24^.

### Trp-cage protein

**Figure 5:**
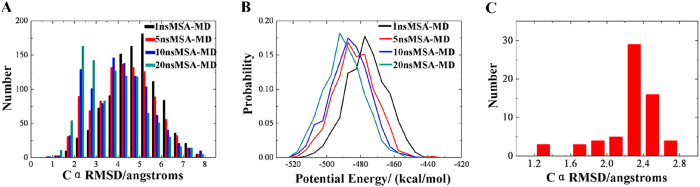
The Cα RMSD and the potential energy distributions of 1, 5, 10, and 20 ns MSA-MD simulations. (**A**) The Cα RMSD distribution of 1, 5, 10, and 20 ns MSA-MD. (**B**) The potential energy distribution of 1, 5, 10, and 20 ns MSA-MD. (**C**) The Cα RMSD distribution of the 64 structures after screening. The distribution of Cα RMSD value of the final 64 structures after screen.

The 1000 folding simulations of the Trp-cage miniprotein were extended to 20 ns. Figure 5A,B shows the Cα RMSD and the potential energy distribution of the 1000 structures extracted from 1, 5, 10, and 20 ns MSA-MD trajectory. Conformations with much lower Cα RMSD values and potential energies were obtained when extending the time scale from 1 to 20 ns, which is different with the simulation of ALPHA1. Hence, the 20 ns MSA-MD simulation is not enough for Trp-cage miniprotein’s folding. Although the distribution of the Cα RMSD and potential energy would be further improved by extending the simulation time, it’s obvious time consuming.

As well known the convergence of the simulation is also dependent on folding process. The simulation in short time scale may also reach a convergence. Hence, a standard deviation (STD) based criteria was introduced to judge the convergence of each MSA-MD simulation. The STD value of the RMSD relative to the initial structure was calculated each 500 ps. If the trajectory is not largely fluctuated (with STD lower than 0.2), the MSA-MD simulation should be terminated and the final structure was extracted or the MSA-MD simulation continue for the next 500 ps until the maximum of 20 ns time scale and no structure will be produced. The mass-weighted RMSD curve is relatively smooth during the 500 ps simulation (shown in Figure S1 in the Supporting Information). Finally the 1000 folding simulations took 14.8850 μs in total and 530 structures were generated which took 5.4145 μs in total after the maximum of 1000 simulation cycles. Based on the distribution of the Cα RMSD of these structures relative to the native structure, the Cα RMSD values of 314 structures (59.25% of the total) are lower than 3 Å and 41 structures (7.74% of the total) are lower than 2 Å.

Then, the 530 structures were screened by the same strategy. First, all of the 530 structures were validated by PROCHECK program and 127 structures (23.96% of the total) with residues in most favored regions more than 80% were screened out. Due to the size of this protein (the size of the Trp-cage protein 1L2Y is almost double to the size of the protein ALAPHA1) and the stereochemical qualities of structures, the RMSD threshold of 3.0 Å was utilized in the next step. The 127 structures were classified into 3 clusters with 78 structures in the largest cluster, which is 61.42% of the total. Thirdly, the terminal residues of the 78 structures were cut and a RMSD threshold of 2.4 Å was utilized. All the above 78 structures were classified into 2 clusters with 64 structures in the largest cluster, which is 82.05% of the total.

Similar with ALPHA1, the screened structures were more and more similar with the native structure of Trp-cage protein. Figure 5C shows the Cα RMSD distribution of the final 64 structures after screening. It is clear that the Cα RMSD values of 64 structures are all lower than 3.0 Å. And there are 10 structures with Cα RMSD lower than 2.0 Å. The best structure (Cα RMSD = 1.284 Å, backbone RMSD = 1.224 Å) predicted by MSA-MD is very close with the previously best structure (backbone RMSD = 1.3 Å) predicted by Neil *et al.*^10^

### PolyAla

**Figure 6:**
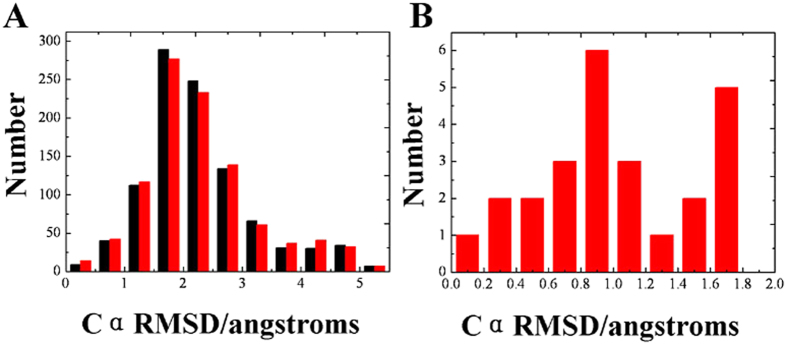
The structures formed by MSA-MD compared with PolyAla fully helical structure. (**A**) The Cα RMSD distribution of the 1000 structures extracted from 10 and 20 ns MSA-MD compared to PolyAla structure. Black represents 10 ns MSA-MD and red represents 20 ns MSA-MD. (**B**) The Cα RMSD distribution of the final 25 structures after screening. The Cα RMSDs of 25 structures are lower than 2.0 Å with 14 structures lower than 1.0 Å, which is 56% of the total.

In order to examine the convergence of the simulated annealing, the potential energy distribution of the final structures from 10 and 20 ns were compared (Fig. 6). As shown in Fig. 6A, there is no significant difference of the Cα RMSD distribution of 10 and 20 ns simulations, which indicates that the 10 ns time scale simulation of the PolyAla is convergent.

Then, the same screening strategy was applied on PolyAla. First, all of the 1000 structures were validated by PROCHECK program and 135 structures (13.50% of the total) with residues in most favored regions more than 80% were screened out. Then, the 135 structures were further classified into 6 clusters according to a threshold RMSD of 1.5 Å using MaxCluster program. The largest cluster of 43 structures is reserved, which occupy 31.85% of the total. Last, excluding the flexile termini residue, a threshold RMSD of 1.0 Å was used, and the 43 structures were further classified into 2 clusters with 25 structures in the largest cluster, which is 58.14% of the total. The screened structures were similar with the native state of PolyAla. Figure 6B shows the Cα RMSD distribution of the final 25 structures. The ratio of Cα RMSD < 1.0 Å and < 2.0 Å is 56.00% and 100% respectively, while the ratio is 4.90% and 45.00% before screening. The Cα RMSD values of all the 25 structures are lower than 2.0 Å while there are 14 structures with Cα RMSD lower than 1.0 Å. The Cα RMSD of the best structure is 0.197 Å compared with the fully helical conformation.

## Conclusion

The MSA-MD method is applied for the structure prediction of ALAPHA1, Trp-cage miniprotein, PolyAla, β sheet structure and miniproteins, which shows good ability in sampling lower energy conformations and searching wider conformational space. A structural ensemble containing 113 structures of ALAPHA1 (Cα RMSD < 2.2 Å), 64 structures of Trp-cage miniprotein (Cα RMSD < 3.0 Å) and 25 structures of PolyAla (Cα RMSD < 2.0 Å) was obtained. Table S1 summarizes the structure prediction results for the representative peptides associated with different methods reported in recent years. Predictive results of these studies are mainly evaluated by RMSD. MSA-MD method turned out to be very efficient in predicting structures close to the native structure. In future work, the method will be extended to the structural prediction of other peptides.

## Methods

### Large scale structural sampling by MSA-MD

The starting structure was a fully extended conformation of the peptide/protein sequence, which was built by Discovery Studio Client 2.5. The topology and coordinate files of the structure were built with Leap module of the Amber12 package. Energy minimization was performed using the Sander module of the Amber12 program. The Amber ff99 force field was used as the parameters for the amino acid residues^43^. Solvation effects were incorporated by using the Generalized Born model^44^. So the simulation was performed in an implicit Generalized Born model. The cutoff distance for the long-range electrostatic interaction and VDW interaction was set at 999.0 Å because this simulation is a non-periodic simulation and the Particle Mesh Ewald (PME) method didn’t need to give infinite electrostatics^45^,^46^. The maximum distance between atoms pairs that will be considered when calculating the effective Born radii was also set at 999.0 Å. Then the structure was subjected to two stages of minimization. First, the backbone atoms of the structure were fixed. Next, all atoms were permitted to move freely. In every stage, the energy minimization was executed by using the steepest descent method for the first 1000 steps and then followed 1000 steps minimization by using the conjugated gradient methods. 1000 simulated annealing (SA-MD) simulations started from the same minimization structure but different initial velocities were used by different trajectories. To prevent unwanted rotations around the peptide bond which might occur leading to non-physical chiralities at high temperature, a chirality restraint on the backbone was used. Next, a simulated annealing process was performed as follows: first, heating the system from 10 K to 500 K in 50 ps; second, a 30 ps production simulation was performed to stable the system; finally, cooling the system to 0 K in 70 ps. The simulated annealing process was performed with the step size of 1fs. Then, the long-time production MD simulation was running from the structure after the simulated annealing process. The system simulated at 300 K by using the weak-coupling algorithm^47^. The simulation process was performed with the step size of 2 fs. All production MD simulations were performed without any restraints.

The convergence of the simulation was examined and the simulated structures was furthered analyzed. If the simulation cannot converge in a limited time scale, a convergence criteria based on RMSD fluctuation would be introduced. The standard deviation (STD) of the RMSD values was calculated each 500 ps. If the STD is lower than the standard values, the simulated will be terminated in limited time scale and the final structure will be produced. If not, the simulation will be prolonged until the largest time scale limitation. We can obtain a large number of predicted structures by performing multiple simulation. In addition, Post-simulation analyses were performed for the determination of residue secondary structural assignments using the DSSP program^25^.

### Structural Screening

To obtain the conformational ensemble similar with the native state, an empirical-based screening strategy was proposed. First, the stereochemical qualities of structures predicted by MSA-MD simulation were evaluated by using PROCHECK program^41^,^42^ and the first 20% structures were screened out. Then the structures passed the PROCHECK screening were further evaluated by using the MaxCluster program. The Nearest Neighbour (NN) clustering algorithm in MaxCluster was used to cluster structures^48^. Two structures are considered in the same cluster, if the backbone RMSD is within an acceptable value. Here, a RMSD cut-off threshold was set to cluster the structures through comparing the RMSD between each other. During the clustering process, the RMSD values between different structures were calculated by using MaxCluster program^49^,^50^. A central structure with the closest neighbourhood with other structures in one cluster group was picked out. The second screening step was roughly performed by setting a relatively bigger cluster threshold. Due to the flexibility, the difference of the Cα RMSD value of the terminal residue may disturb the cluster. In order to strike off this influence, another cluster analysis to the structures from the largest cluster groups was performed. In this step, the RMSD values of the terminal residues were not taken into account and a relatively small cluster threshold was used. Finally, the structures of the largest cluster group in the third step were the targeted structures.

## Additional Information

**How to cite this article**: Hao, G.-F. *et al.* Multiple Simulated Annealing-Molecular Dynamics (MSA-MD) for Conformational Space Search of Peptide and Miniprotein. *Sci. Rep.* **5**, 15568; doi: 10.1038/srep15568 (2015).

## Supplementary Material

Supplementary Information

## Figures and Tables

**Figure 1:**
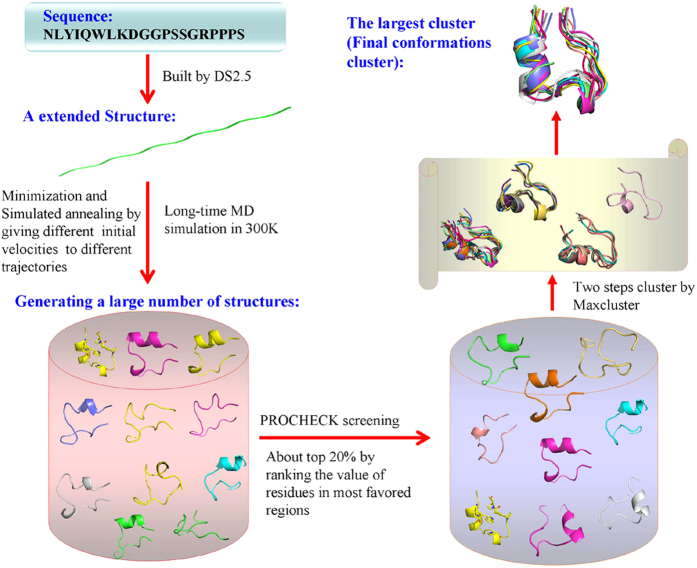
A flowchart of Multiple Simulated Annealing-Molecular Dynamics (MSA-MD) to predict the structures of small peptides. It shows the prediction process of small peptides structures in details. First, a full extended conformation of the peptide was built by Accelrys Discovery Studio2.5 from the primary sequence. Second, large scale structure sampling was performed through energy minimization, simulating annealing, and refined MD simulation to produce a large number of structures. Third, the stereochemical qualities of those structures were evaluated and about 20% of the total is screened out. Fourth, the structures with better stereochemical qualities were screened by clustering.

**Table 1:** Comparison of the results from MSA-MD simulation of the seven peptides.

**Protein**	**Chain length**	**Secondary structure type**	**time scale (ns)**	**RMSD region (residue numbers)**	**Lowest Cα RMSD/Å**
1AL1	12	α-helix	10	1–12	0.198
1L2Y	20	α-helix	10	1–20	0.960
PolyAla	11	α-helix	10	1–11	0.197
1UAO	10	β–turn	10	1–10	1.200
1E0Q	17	β–sheet	10	1–17	2.955
1ERD	40	α-helix	10	4–34	2.908
1GAB	53	α-helix	10	9–52	4.715
