# Supplementary material for: Multiple Simulated Annealing-Molecular Dynamics (MSA-MD) for Conformational Space Search of Peptide and Miniprotein
Source: Sci Rep. 2015 Oct 23;5:15568. doi: 10.1038/srep15568 (PMC4616061; doi:10.1038/srep15568)
Supplement: Supplementary Information [file srep15568-s1.doc]

**Supporting Information**

# Multiple Simulated Annealing-Molecular Dynamics (MSA-MD) for Conformational Space Search of Peptide and Miniprotein

Ge-Fei Hao,^a^,*Wei-Fang Xu,^a^ Sheng-Gang Yang,^a^ and Guang-Fu Yang^a,b^,*

*^a^Key Laboratory of Pesticide & Chemical Biology, Ministry of Education, College of Chemistry, Central China Normal University, Wuhan 430079, P.R.China; ^b^Collaborative Innovation Center of Chemical Science and Engineering, Tianjing 300072, P.R.China*

**Correspondence:**

Guang-Fu Yang, Ph.D. & Professor

College of Chemistry

Central China Normal University

152 Luoyu Road

Wuhan, Hubei, P. R. China 430079

TEL: 86-27-67867800

FAX: 86-27-67867141

E-mail: [gfyang@mail.ccnu.edu.cn](mailto:gfyang@mail.ccnu.edu.cn)

—————————

*To whom correspondence should be addressed. *E-mail:* [*gfyang@mail.ccnu.edu.cn*](mailto:gfyang@mail.ccnu.edu.cn)*;* [*gfhao@mail.ccnu.edu.cn*](mailto:gfhao@mail.ccnu.edu.cn)

**Figure S1.** The time course of the mass-weighted RMSD relative to the MD initial structures during the 500ps simulation of a stage (the 25th stage of No.99 structure which is a representative structure passed the convergent criteria) whose standard deviation (STD) is lower than 0.2. It indicates that the fluctuation of the structure is small and the mass-weighted RMSD curve is relatively smooth during the 500ps equilibration stage. So, in this stage whose standard deviation (STD) is lower than 0.2, the trajectory is thought to be convergent and there is no need to continue the next equilibration simulation stage.

Table S1 Comparison the prediction results of ALPHA1 and Trp-cage miniprotein by different simulation methods.

| Protein | Method | Result | Year |
| --- | --- | --- | --- |
| ALPHA1 | SA-REMD | Heavy atoms RMSD = 1.3Å. | 2009^1^ |
|  | MSA-MD | Cα RMSD = 0.3Å  Heavy atoms RMSD = 0.9Å. |  |
| Trp-cage miniprotein | SMD | CαRMSD = 2.1 Å. | 2002^2^ |
|  | REMD | Heavy atom RMSD = 2.0 Å | 2009^2^ |
|  | CSMD | Backbone RMSD = 1.3 Å. | 2010^3^ |
|  | Cons-MD | Backbone RMSD = 1.9 Å | 2011^4^ |
|  | MSA-MD | Cα RMSD = 1.3Å  Backbone RMSD=1.2Å |  |

SA-REMD: simulated annealing coupled replica exchange molecular dynamics

MSA-MD: multiple simulated annealing molecular dynamics

SMD: stochastic molecular dynamics

REMD: replica exchange molecular dynamics

CSMD: a cooperative swarm of molecular dynamics trajectories

Cons-MD: constrained molecular dynamics

**Reference：**

1. Kannan, S. & Zacharias, M. Simulated annealing coupled replica exchange molecular dynamics--an efficient conformational sampling method. *J Struct Biol* **166**, 288-294 (2009).

2. Snow, C.D., Zagrovic, B. & Pande, V.S. The Trp cage: Folding kinetics and unfolded state topology via molecular dynamics simulations. *J Am Chem Soc* **124**, 14548-14549 (2002).

3. Bruce, N.J. & Bryce, R.A. Ab Initio Protein Folding Using a Cooperative Swarm of Molecular Dynamics Trajectories. *J Chem Theory Comput* **6**, 1925-1930 (2010).

4. Balaraman, G.S., Park, I.H., Jain, A. & Vaidehi, N. Folding of small proteins using constrained molecular dynamics. *J Phys Chem B* **115**, 7588-7596 (2011).
